# Service availability and association between *Mutuelles* and medical care usage for under-five children in rural Rwanda: a statistical analysis with repeated cross-sectional data

**DOI:** 10.1136/bmjopen-2015-008814

**Published:** 2015-09-08

**Authors:** Iván Mejía-Guevara, Kenneth Hill, S V Subramanian, Chunling Lu

**Affiliations:** 1 Harvard Center for Population and Development Studies, Harvard University, Cambridge, Massachusetts, USA; 2 Department of Global Health and Population, Harvard T.H. Chan School of Public Health, Boston, Massachusetts, USA; 3 Department of Social and Behavioral Sciences, Harvard T.H. Chan School of Public Health, Boston, Massachusetts, USA; 4 Division of Global Health Equity, Brigham & Women's Hospital, Boston, Massachusetts, USA; 5 Department of Global Health and Social Medicine, Harvard Medical School, Boston, Massachusetts, USA

## Abstract

**Objective:**

To compare the association between *Mutuelle*s enrolment and medical care utilisation among under-five rural children between 2005 and 2010; that is, before and after substantial improvements in service availability took place in rural areas.

**Methods:**

We tracked the change in service availability between 2005 and 2010. Using the nationally representative population-based Rwanda Demographic and Health Surveys 2005 and 2010, we conducted a statistical analysis using multilevel logistic random-effects models. We included *Mutuelles* enrollees and uninsured children who had diarrhoea, cough or fever in the previous 2 weeks of the surveys. The final sample size was 4071 children.

**Results:**

We observed a substantial increase in the availability of health facilities, medical staff and child health services from 2005 to 2010. In both years, under-five children with *Mutuelles* were more likely to use medical care than uninsured children. Children in 2010 had a higher probability of using medical care than their counterparts in 2005, regardless of the children's poverty or *Mutuelles* status. *Mutuelles* enrollees in 2010 had the highest probability of using care among children in both years. The findings were robust to model specifications and estimation methods.

**Conclusions:**

This study suggests the importance of strengthening service provision at the supply side in promoting equitable utilisation of childcare with prepayment schemes.

**Strengths and limitations of this study**

- Previous studies have neither considered clustering effects nor captured the effects of a series of policy programmes on strengthening medical care provision after 2006. We addressed clustering effects in the statistical analysis.
- We identified the effects of strengthening healthcare delivery by comparing the association between *Mutuelles* enrolment and use of medical care for under-five rural children in 2005 with that in 2010 when service provision was much improved.
- With cross-sectional data, we were unable to identify causal effects between *Mutuelles* participation and medical care provision for under-five children.

## Introduction

To promote equitable access to child health services in resource-poor settings, programmes such as conditional cash transfers and community health insurance have been implemented to assist poor households in overcoming financial barriers to access care. Concerns have been raised on inadequate attention to service provision.^1^ After all, without sufficient quality services, it is difficult to meet the increased demand for childcare.

*Mutuelles*, a community-based health financing programme in Rwanda, was designed to (1) reduce the financial burden for enrolled households, and (2) mobilise domestic resources to support the Minimum Service Package (MSP) or the Complementary Service Package provided by health centres or hospitals (box 1). Unlike conditional cash transfers whose main focus is on increasing the demand for services, the *Mutuelles* programme addresses financial bottlenecks on the demand and supply sides. While it offers prepayment insurance to the rural households, *Mutuelles* also pays rural health centres for providing MSP (box 1). To date, about 91% of the population in Rwanda participated in the programme.^2^

Box 1 **Child health services covered by *Mutuelles* in Rwanda**

*Mutuelles* was piloted in 1999 and scaled up to the national level since 2006.

Premium and copayments

Enrolling in *Mutuelles* is voluntary. The subscription unit is the household. Between 2006 and 2010, each household paid a premium of 1000 Rwandan Franc (US$1·8) per member, 200 Rwandan Franc (US$0.36) copayments for each health centre visit, and 10% of the hospital fee for hospitalisation. Since 2011, the poorest population (about 25%) was fully subsidised (exempted from paying premiums and copayments) by the government and donors.^2^

Child health services included in the Minimum Service Coverage (MSP)*

In 2008, a law for the “creation, organisation, functioning and management” of *Mutuelles* was enacted, defining a Minimum Service Package provided by health centres and a Complementary Service Package provided by hospitals. The *Mutuelles* programme collects and transfers risk-pooling funds to public health facilities on a regular basis to cover the expenses related to the provision of MSP to *Mutuelles* enrollees.^2^ ^3^ Our analysis on Rwanda health facility data shows that funds obtained from the *Mutuelles* programme accounted for about 22% of the total funds received by rural health centres in 2010, and this provides a long-term financial support to health facilities for the provision of all services under the MSP, which include medical care services for under-five children. The services covered by the MSP are categorised as follows:

1. Promotional and educational services (5)

Campaigns on malaria, vaccinations, nutrition, insecticide**-treated** bed **nets** (ITN) and integrated management of childhood illness (IMCI).

2. Preventive care (16)

Child vaccination: polio 2-DTC-HepB/Hib2, Bacille Calmette-Guerin (BCG), polio 3-DTC-HepB/Hib3, polio 0, polio 1-DTC-HepB/Hib1, measles, pneumococcal, rubella, mumps; malnutrition screening for children under age 5 (weight and/or MUAC); vitamin A supplements for children; growth monitoring for children: weight-for-age (WAZ), height-for-age (HAZ); neonatal service of breastfeeding support; feeding programmes in school; health monitoring in school.

3. Curative care (15)

Providing amoxicillin/mebendazole/vitamin A/vaccine/folic acid/HIV care/iron/tuberculosis (TB) care to children with severe malnutrition; oral rehydration salts for patients with diarrhoea; neonatal service of post-eutocic delivery care/resuscitation/suction and bathing/sepsis treatment; newborn check-ups; inpatient food support to children.

*Sources: District Health System Strengthening Tool 2010.

Previous studies have found a significant effect of *Mutuelles* on individual enrollees’ utilisation of medical care using the Rwanda Demographic and Health Survey (RDHS) 2005.^4–6^ Those studies, however, have two limitations. First, they did not address clustering effects. The RDHS was collected through a two-stage sampling process. Individual children from the same sampling unit could be more similar to each other than those from other clusters. In addition, since 2006, Rwanda has been decentralising healthcare delivery to its 30 administrative districts. Children are clustered within district health systems. Ignoring the clustering effects at the sampling unit and district levels may lead to conclusions on a relationship that actually does not exist.^7^ Second, the health sector context changed drastically between 2005 and 2010. As *Mutuelles* was implemented at the national level in 2006, many important policy measures, such as pay-for-performance (PFP) and minimum service package (MSP) of *Mutuelles* at the health centres,^4^ ^8^ were rolled out to strengthen healthcare delivery. Previous studies were not able to capture *Mutuelles*’ effect in the new context. We are not aware of publications that have used the most recent data (RDHS 2010) to address these two issues.

Taking advantage of the repeated cross-sectional data RDHS 2005 and 2010, our study investigated the association between *Mutuelles* enrolment and medical care utilisation for under-five rural children with diarrhoea and respiratory infections. We examined the change in Rwanda's healthcare sector between 2005 and 2010 using information from government reports from Rwanda,^3^ ^9–13^ and paid special attention to compare the association in 2005 with that in 2010. We addressed the clustering effects and also explored the effect of social demographic factors on medical care utilisation.

## Methods

### Data sources

To track the change in health service availability in Rwanda between 2005 and 2010, we gathered available data on health sector financing, as well as the number of health facilities and medical staff from sources such as the Annual Report of Rwanda Ministry of Health, Rwanda Health Statistics, Rwanda Statistical Yearbook and National Health Accounts produced by the WHO.^3^ ^9–13^ We used the District Health System Strengthening Tool (DHHST), a web-based database built by the Ministry of Health in Rwanda for monitoring and strategic planning on health systems strengthening,^13^ to study the availability of child health services covered by the MSP of *Mutuelles* across rural health centres. The database only provided information about medical service provision across health centres since 2009, and we were therefore not able to obtain information about service provision in 2005.

The RDHS was used for conducting individual-level analysis. It is a nationally representative population-based survey that has been conducted every 5 years to measure the indicators of population health with a specific emphasis on mothers and under-five children. The RDHS collects information on the child, mother and household’s sociodemographic indicators, health insurance status, utilisation of healthcare, etc.^14^ It is publicly accessible at https://dhsprogram.com/Data/.

### Study population and sample size

The target population for this study was the under-five children in rural Rwanda who had diarrhoea, cough or fever in the previous 2 weeks of the surveys. To analyse the association between *Mutuelles* enrolment and childcare utilisation, we only included under-five children in the RDHS who were either enrolled in the *Mutuelles* or had no insurance. We deleted data of children with other types of insurance, which was about 1.4% in 2005 and 2.2% in RDHS 2010. We excluded children who received the treatment from traditional healers or friends or relatives, and dropped 807 observations in 2005 and 233 in 2010. The final sample size was 2209 children in 2005 and 1862 in 2010.

### Variables

#### Outcome

We constructed a dichotomous variable to indicate whether a child received medical care from health facilities if she had diarrhoea, fever or cough in the previous 2 weeks of the survey.

#### Exposure

A dichotomous variable ‘*Mutuelles’* was constructed to indicate whether an under-five child was enrolled in *Mutuelles* or uninsured in 2005 and 2010.

Since 2006, the Government of Rwanda has adopted a series of policy measures to strengthen health sector financing and improve the availability and quality of health services. Health centres in Rwanda were required to provide a series of child health services covered by the MSP of the *Mutuelles* programme. The services range from promotional, to preventive, to curative care (box 1). Community health workers were trained to conduct community integrated management of childhood illnesses (C-IMCI) in rural Rwanda and treated diarrhoea, fever and pneumonia during their home visits.^12^ In addition, the PFP scheme provides economic incentives to health facilities for providing quality services, including child and maternal care.^8^ To capture the change of service delivery between 2005 and 2010, we constructed a year indicator variable defined as 1 for the year 2010 and 0 otherwise. To examine the difference in the association between *Mutuelles* and childcare utilisation under different service delivery contexts in 2005 and 2010, we constructed an interaction variable between *Mutuelles* and year indicators.

#### Covariates

Sociodemographic variables included child characteristics (age and gender), maternal characteristics (mother’s age and education) and household characteristics (poverty status, geographic difficulty to access the nearest health facilities). Child's age was defined dichotomously as 1 for ages between 24 and 59 m, and 0 otherwise. Two dichotomous variables were constructed to indicate whether a child’s mother completed primary school or was 30 or older. A poverty indicator suggests whether a child was from a household living under the poverty line, defined as the households in the first, second and third wealth quintiles in the RDHS 2005 and the first and second wealth quintiles in 2010. The construction of the poverty indicator was based on the fact that about 57% and 45% of rural households lived under the poverty line in 2005 and 2010, respectively.^1^ A dichotomous variable was constructed to indicate whether a household reported geographic difficulty to access the nearest health facilities. To examine *Mutuelles*’ effect on improving disparities in utilisation between the poor and non-poor households, we constructed an interaction variable between poverty and *Mutuelles* coverage.

The RDHS collected data in two stages. In the first stage, villages or primary sampling units (PSUs) were selected, and households were selected in the second stage from the previously selected villages. Village-level government agencies played an important role in establishing and promoting *Mutuelles*. To control for the variation of village-level efforts in public health across the PSU, we constructed a variable indicating the enrolment rate of *Mutuelles* at the PSU level and included it in the individual level analysis.

### Ethics approval

We used the RDHS 2005 and 2010, publicly accessible data, to conduct secondary data analysis in this study. The identity of the subjects has been removed and does not require further ethical considerations.

## Statistical analysis

The analysis was executed at the national and rural-individual levels. At the national level, we traced the temporal trends of health sector financing and the expansion of health facilities, medical staff and service availability between 2005 and 2010. At the individual level, we investigated the association between a rural child’s *Mutuelles* enrolment and her utilisation of medical care in 2005 and 2010 using the repeated cross-sectional data RDHS 2005 and 2010.

For individual-level analysis, we used a multilevel logistic random-effects model which allowed us to control for clustering effects at the PSU and district levels. A three-level multilevel model was used to capture four sources of variation in the probability analysis: individual child and her mother and household characteristics, PSU factors, district-level factors and random errors. A standard two-step procedure for discrete outcomes multilevel models was used for random-effects estimation.^7^ ^15^ Details are presented in the online supplementary material. We reported ORs and credible intervals (CrI) which indicate that the true estimate lies in the CrI with a probability of 95%.^16^ ^17^ STATA 12 and MLwiN 2.28 were used in the statistical analysis.^7^ ^16^

Adjusting for cluster effects and other covariates, we estimated (1) the main association between childcare utilisation and year and *Mutuelles* indicators (model 1 in table 1), (2) the association between *Mutuelles* and childcare utilisation for children in different years with an interaction variable between *Mutuelles* and year indicators (model 2 in table 1), and (3) the association between *Mutuelles* and childcare utilisation for children in different years and poverty status with interactions between *Mutuelles* and year indicators and between *Mutuelles* and poverty status (models 3 at table 1).

**Table 1:** Multilevel models with different model specifications

Main association	
Model 1	F=f(*Mutuelles*, year, poverty, child age, child gender, mother's education, mother's age, geographic difficulty, PSU-*Mutuelles*)
Association by year and *Mutuelles*	
Model 2	F=f(*Mutuelles*, year, Mutuelles X year, child age, child gender, mother's education, mother's age, geographic difficulty, PSU-*Mutuelles*)
Association by year and poverty status	
Model 3	F=f(*Mutuelles*, year, Mutuelles X year, poverty, Mutuelles X poverty, child age, child gender, mother's education, mother's age, geographic difficulty, PSU-*Mutuelles*)

PSU, primary sampling unit.

We tested the sensitivity of results to different estimation methods using a fixed-effects model by including district indicators in the individual-level analysis.^18^

## Results

### Health facilities, medical staff and child health services between 2005 and 2010

The Rwandan National Health Accounts show that health expenditure as a percentage of GDP in Rwanda has increased sharply from 6.1% in 2005 to 10.8% in 2010 (table 2). Increased investment in the health sector made it possible to expand health facilities, medical staff and service availability. The number of health facilities has steadily increased from 353 health centres in 2005 to 436 in 2010. The number of physicians and nurses in 2010 (604 and 8202) was almost double of that in 2005 (309 and 4989). Data about community health workers in 2005 are not available. The available data show that community health workers increased approximately from 45 000 in 2008 to 60 000 in 2011 (table 2).

**Table 2:** Health sector indicators between 2005 and 2010

	Health spending as % of GDP	District hospitals	Health centres	Physicians	Nurses/midwife	Community health workers
2005	6.1	38	353	309	4989	NA
2008	10.0	40	406	530	7088	45 000
2010	10.8	40	436	604	8202	60 000 (2011)

Source: Rwanda Ministry of Health.^3^ ^9–11^ ^13^

GDP, gross domestic product; NA, not available.

Table 3 presents the percentage of available child health services provided by 390 rural health centres in 2010. A high percentage of them offered promotional or educational services, including campaigns on malaria and vaccines (98%), nutrition (94%), insecticide-treated bed nets (86%) and IMCI (78%). Almost all of the rural health centres offered vaccination and malnutrition screening for under-five children, and around 90% of facilities provided vitamin A supplements and growth monitoring for children. High coverage was also observed in the provision of a few curative services, mainly for severe malnourished children (mebendazole, vitamin A, vaccine, HIV care, oral rehydration salts for diarrhoea) and neonatal care (post-eutocic delivery care and neonatal resuscitation).

**Table 3:** Percentage of rural health centres with available child health services in 2010 (N=390)

	Per cent
(1) Promotional services (6)	
Campaign on malaria	97.7
Campaign on vaccinations	97.7
Campaign on nutrition	93.6
Campaign on ITN	85.9
Campaign on integrated management of childhood illness	77.9
(2) Preventive care (16)	
Child vaccination of polio 2-DTC-HepB/Hib2	99.2
Child vaccination of BCG	99.0
Child vaccination of polio 3-DTC-HepB/Hib3	99.0
Child vaccination of polio0	98.7
Child vaccination of polio 1-DTC-HepB/Hib1	98.7
Child vaccination of measles	97.7
Child vaccination of pneumococcal	97.4
Child vaccination of rubella	6.2
Child vaccination of mumps	4.6
Malnutrition screening for under-5 children (weight and/or MUAC)	95.4
Vitamin A supplements for children	92.6
Growth monitoring for children: weight-for-age	89.2
Growth monitoring for children: height-for-age	89.2
Neonatal service of breastfeeding	74.6
Feeding programmes in school	35.9
Health monitoring in school	12.8
(3) Curative care (15)	
Providing amoxicillin to children with severe malnutrition	37.9
Providing mebendazole to children with severe malnutrition	88.7
Providing vitamin A to children with severe malnutrition	85.1
Providing vaccine to children with severe malnutrition	82.8
Providing folic acid to children with severe malnutrition	45.4
Providing HIV care to children with severe malnutrition	75.6
Providing iron to children with severe malnutrition	47.7
Providing TB care to children with severe malnutrition	53.6
Distribution of oral rehydration salts for patients with diarrhoea	70.5
Neonatal service of post-eutocic delivery care	92.3
Neonatal resuscitation	65.4
Neonatal service of suction and bathing	29.0
Neonatal sepsis treatment	22.3
Newborn check-ups	62.3
Inpatient food support to children	44.1

Source: District Health System Strengthening Tool 2011.

BCG, Bacille Calmette-Guerin; INT, insecticide-treated bed nets; TB, tuberculosis.

### Association between the *Mutuelles* enrolment and medical care utilisation in 2005 and 2010

Definition and summary statistics of variables used in individual-level regression are presented in online supplementary table S1. Results of t tests on the mean difference of medical care utilisation by *Mutuelles* status and covariates are presented in online supplementary table S2, and on the mean difference of *Mutuelles* enrolment by sociodemographic factors in online supplementary table S3.

Online supplementary table S1 illustrates that 44 and 78% of the under-five children were enrolled in *Mutuelles* in 2005 and 2010, respectively. For under-five rural children who had diarrhoea, fever or cough in the previous 2 weeks of the two surveys, 24% and 39% of them used medical care in 2005 and 2010, respectively.

We examined differences in sociodemographic factors between children enrolled in *Mutuelles* and those without any insurance in 2005 and 2010, and found that children living under the poverty line were more likely to be uninsured in both years, in contrast to children with more educated mothers who were more likely to be insured in both years. We also found that children with geographic difficulty to access the nearest health facility were more likely to be uninsured in 2010 (see online supplementary table S3).

Using a covariate adjusted multilevel random-effects logistic model, the main association between medical care utilisation and the year indicator was 1.585 (95% CrI 1.272 to 1.954) (model 1 in table 4), suggesting that holding everything else constant, children in 2010 were more likely to use medical care than children in 2005. The main association between *Mutuelles* enrolment and using medical care after adjusting for covariates was positive and statistically significant (1.758, 95% CrI 1.447 to 2.100) (model 1 in table 4).

**Table 4:** Estimated OR of year and *Mutuelles* indicators from several model specifications using multilevel random-effects logistic regression models with pooled 2005 and 2010 data

	OR with 95% CrI
Model 1* (main association between medical care use and 2010 year indicator)	1.585 (1.272 to 1.954)
Model 1 (main association between *Mutuelles* and medical care use)	1.758 (1.447 to 2.100)
Model 2 (association between *Mutuelles* and medical care use in 2005)	1.645 (1.303 to 2.044)
Model 2 (association between *Mutuelles* and medical care use in 2010)	1.947 (1.469 to 2.582)
Model 3 (association between *Mutuelles* and medical care use for children in 2005 and under the poverty line)	1.693 (1.308 to 2.193)
Model 3 (association between *Mutuelles* and medical care use for children in 2005 and above the poverty line)	1.541 (1.121 to 2.093)
Model 3 (association between *Mutuelles* and medical care use for children in 2010 and under the poverty line)	2.021 (1.497 to 2.730)
Model 3 (association between *Mutuelles* and medical care use for children in 2010 and above the poverty line)	1.816 (1.282 to 2.574)
N	4071

*For definition of model 1 to model 3, please refer to table 1.

The OR of *Mutuelles* in 2010 (1.947, 95% CrI 1.469 to 2.582) was larger than that in 2005 (1.645, 95% CrI 1.303 to 2.044) (model 2 in table 4). For children in different survey years and poverty groups, the OR of *Mutuelles* was the largest for children living in poverty in 2010 (OR: 2.021; 95% CrI 1.497 to 2.730). Children above the poverty line in 2010 had the second largest OR of *Mutuelles* (1.816; 95% CrI 1.282 to 2.574). Children living in poverty in 2005 ranked third (OR: 1.693; 95% CrI 1.308 to 2.193). Children above the poverty line in 2005 had the lowest estimate of *Mutuelles* (OR: 1.541; 95% CrI 1.121 to 2.093) (model 3 in table 4).

Other significant factors associated with using medical care included a child’s age, poverty status, mother’s education and geographic difficulty to the nearest health facilities (see online supplementary table S4).

**Figure 1:**
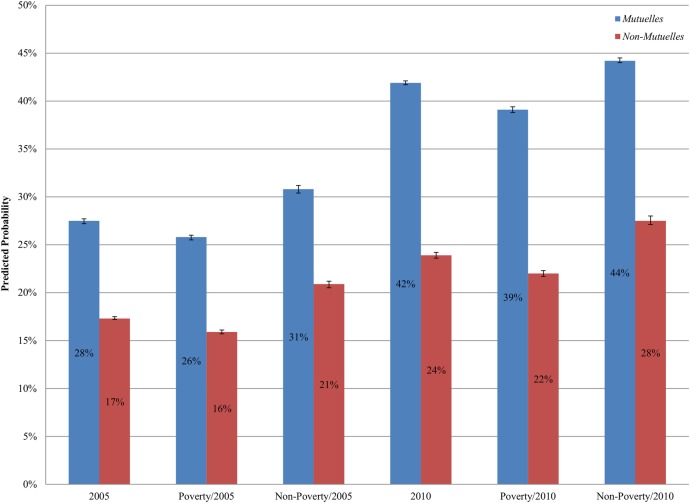
Predicted probability of obtaining care when having diarrhoea, fever or cough using multilevel random-effects logistic models.

We generated predicted probabilities of using care for children from different survey years and/or poverty status, controlling for other covariates and multilevel random effects (figure 1). Compared to children in 2005, the probability of receiving care for both insured and uninsured children in 2010 was significantly higher: 42% (2010) versus 28% (2005) for *Mutuelles* enrollees, and 24% (2010) versus 17% (2005) for uninsured children (figure 1). This result stayed unchanged when considering children’s poverty status: for children in different poverty groups, both *Mutuelles* enrollees and uninsured children in 2010 had a higher probability of using care than children in 2005. For example, for children living below the poverty line in 2005, the probability of using medical care was 25.8% (95% CI 25.6% to 26.1%) for *Mutuelles* enrollees and 15.9% (95% CI 15.7% to 16.1%) for the uninsured. For children who lived below the poverty line in 2010, the probability of using medical care was 39.1% (95% CI 38.8% to 39.4%) for *Mutuelles* enrollees and 22.0% (95% CI 21.7% to 22.3%) for the uninsured (figure 1). For each group of children, *Mutuelles* enrollees had a significantly higher probability of using medical care than uninsured children when they were ill.

Our estimates on clustering effects reveal that most of the between group variation of medical care treatment (84%) was attributable to PSUs rather than to districts, suggesting that the correlation of medical treatment among children living in the same village and same district was substantially higher than the one for children living in different villages within the same district (see the online supplementary material for details on the estimation method).

### Sensitivity tests

We compared the OR estimates of year and *Mutuelles* indicators derived from the random-effects and fixed-effects model and found that the estimates from these two models were consistent and very close in magnitude (see online supplementary table S5). For example, the OR for the 2010 year indicator derived from the fixed-effects model was 1.545 (95% CI 1.254 to 1.905), very close to that in the random-effects model. This suggested that the estimates of year and *Mutuelles* are not sensitive to estimation methods.

## Discussion

Using pooled RDHS 2005 and 2010 and controlling for clustering effects and other covariates, we have two salient findings. First, rural children with *Mutuelles* were more likely to use medical care than those uninsured when they had diarrhoea, cough or fever. This observation holds for children in 2005 and 2010. Second, children in 2010, the year with a much more strengthened health service delivery system, had a higher probability of using medical care than their counterparts in 2005, regardless of the children's poverty status or *Mutuelles* status. The findings were robust to model specifications and estimation methods.

The positive association between *Mutuelles* enrolment and using medical care for under-five children is consistent with previous studies on *Mutuelles*.^4–6^ Contrary to findings in countries such as Ethiopia in which the poor seemed to be less affected by the community-based health insurance programme than the rich in their medical care utilisation,^19^ *Mutuelles* in Rwanda benefited the enrollees living under the poverty line to the same extent as the enrollees living above the poverty line, if not more. This could be a result from the still ongoing efforts of the Government of Rwanda, with support from donors such as the Global Fund to Fight AIDS, Tuberculosis and Malaria,^20^ in getting the poorest households exempted from premiums.

The observed positive association between the year 2010 and childcare utilisation could be a result of improved service availability and quality delivered in rural health centres and is consistent with existing evidence in India. By comparing the rates of institutional birth deliveries across programmes with no interventions, demand-side interventions only, and interventions on the demand and supply sides, a study in one province of India observed a substantial increase in the delivery rates when introducing interventions on the demand and supply sides.^1^ Since 2006, the Government of Rwanda has been carrying out a wide range of initiatives and interventions at the national and community level to improve child and maternal health. The increased availability and quality of child health services in rural areas is likely to lead to an increase in the likelihood of using the care.

Even with strengthened service provision in health centres, among *Mutuelles* enrollees the utilisation rate was still low in 2010 (42%). There are two plausible explanations. First, *Mutuelles* copayments (200 Rwandan Franc) could still pose a financial burden to rural households living under the poverty line (175 Rwandan Franc per adult per day). We expect to see a substantial increase in medical care utilisation after 2011 when the poorest households are exempted from both premiums and copayments. Second, other sociodemographic factors, such as mother’s education and geographic difficulty for accessing the nearest health facilities, are significantly associated with the utilisation of medical care, suggesting the importance of enhancing women's education and increasing physical accessibility in improving childcare.

One limitation of this study was that we were not able to identify the causal effect of *Mutuelles* on childcare utilisation with cross-sectional data. The repeated cross-sectional data, however, did enable us to investigate the association between *Mutuelles* and medical care utilisation under different settings of service delivery.

By addressing the methodological issues such as clustering effects at the different levels, this study reinforced the findings about the positive association between *Mutuelles* and medical care utilisation among under-five children regardless of their poverty status. The study demonstrated that after strengthening service provision in rural health centres, rural children were more likely to use the care. As a community-based health financing programme, the design of *Mutuelles* provided financial support to the demand and supply sides. Given that the community-based health financing approach has been implemented in other countries in sub-Saharan Africa such as Mali, Senegal, Nigeria and Uganda,^21–25^ knowledge gained from this study may be of great interest to policymakers in these countries.
